# *N*-(6-Methyl-2-pyrid­yl)formamide

**DOI:** 10.1107/S1600536809053549

**Published:** 2009-12-16

**Authors:** Hui-Ling Hu, Chia-Jun Wu, Pei-Chi Cheng, Jhy-Der Chen

**Affiliations:** a Department of Chemical Engineering and Material Engineering, Nanya Institute of Technology, Chung-Li, Taiwan; b Department of Chemistry, Chung-Yuan Christian University, Chung-Li, Taiwan

## Abstract

The mol­ecule of the title compound, C_7_H_8_N_2_O, is essentially planar with a maximum deviation of 0.0439 (1) Å from the best plane. In the crystal, N—H⋯O hydrogen bonds between self-complementary amide groups join mol­ecules into centrosymmetric dimers.

## Related literature

For the synthesis of the title compound, see: Hosmane *et al.* (1984 ▶). For background to this work, see: Wang *et al.* (2006 ▶). For the structure of 2-pyridylformamide, see: Bock *et al.* (1996 ▶).
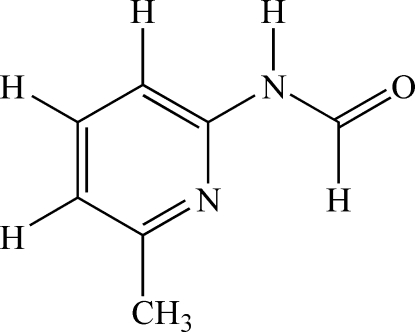


## Experimental

### 

#### Crystal data

C_7_H_8_N_2_O
*M* *_r_* = 136.15
Triclinic, 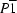

*a* = 4.0611 (6) Å
*b* = 8.6232 (12) Å
*c* = 10.3231 (12) Å
α = 87.421 (12)°
β = 79.344 (14)°
γ = 83.103 (15)°
*V* = 352.61 (8) Å^3^
*Z* = 2
Mo *K*α radiation
μ = 0.09 mm^−1^
*T* = 295 K
0.5 × 0.2 × 0.1 mm

#### Data collection

Bruker P4 diffractometer
Absorption correction: ψ scan(*XSCANS*; Siemens, 1995 ▶) *T*_min_ = 0.713, *T*_max_ = 0.940
1757 measured reflections
1222 independent reflections
993 reflections with *I* > 2σ(*I*)
*R*_int_ = 0.031
3 standard reflections every 97 reflections
intensity decay: none

#### Refinement

*R*[*F* ^2^ > 2σ(*F* ^2^)] = 0.050
*wR*(*F* ^2^) = 0.148
*S* = 1.05
1222 reflections
92 parameters
H-atom parameters constrained
Δρ_max_ = 0.15 e Å^−3^
Δρ_min_ = −0.16 e Å^−3^

### 

Data collection: *XSCANS* (Siemens, 1995 ▶); cell refinement: *XSCANS*; data reduction: *SHELXTL* (Sheldrick, 2008 ▶); program(s) used to solve structure: *SHELXS97* (Sheldrick, 2008 ▶); program(s) used to refine structure: *SHELXL97* (Sheldrick, 2008 ▶); molecular graphics: *SHELXTL*; software used to prepare material for publication: *SHELXTL*.

## Supplementary Material

Crystal structure: contains datablocks I, global. DOI: 10.1107/S1600536809053549/gk2247sup1.cif

Structure factors: contains datablocks I. DOI: 10.1107/S1600536809053549/gk2247Isup2.hkl

Additional supplementary materials:  crystallographic information; 3D view; checkCIF report

## Figures and Tables

**Table 1:** Hydrogen-bond geometry (Å, °)

*D*—H⋯*A*	*D*—H	H⋯*A*	*D*⋯*A*	*D*—H⋯*A*
N1—H1*A*⋯O^i^	0.86	2.04	2.8971 (19)	172

Symmetry code: (i) 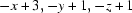.

**Fig. 1.**
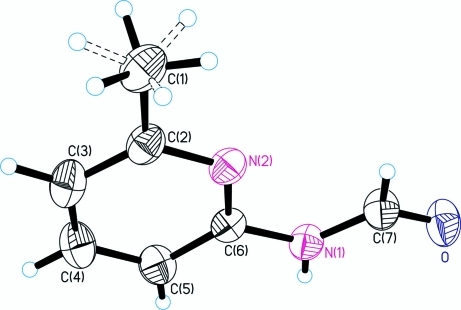
Molecular structure of the title compound with atom labeling and displacement ellipsoids drawn at the 30% probability level. The disorder is shown with open bonds.

## supplementary crystallographic information

### Comment

A series of Ag(I) coordination polymers containg 2-aminopyrimidine or 2-amino-4,6-dimethylpyrimidine ligands have been prepared, which show one-dimensional and two-dimensional structures (Wang, *et al.*, 2006) with interesting bonding modes. To investigate the effect of flexibility of the ligand on the structural type of such coordination polymers, we have synthesized the title compound. Within this project its crystal structure was determined.

The title molecule is almost planar (Fig. 1). In the crystal structure weak intermolecular N—H···O hydrogen bonding is found between self-complementary amide groups (Table 1) that connects molecules into centrosymmetric dimers. In 2-pyridylformamide the molecules formed dimers via hydrogen bonds between self-complementary 2-pyridylamino groups (Bock *et al.*, 1996).

### Experimental

The title compound was prepared according to a procedure reported for *N*-(2-pyrimidinyl)formamide by Hosmane *et al.* (1984). Coloress plate crystals suitable for X-ray crystallography were obtained by dissolving the title compound in CH_2_Cl_2_, followed by allowing the solution to evaporate slowly under air.

### Refinement

All the hydrogen atoms were placed into idealized positions and constrained by the riding atom approximation with *C*—H = 0.93 — 0.96 Å, N—H = 0.86 Å and *U*_iso_(H) = 1.5 *U*_eq_(C) or 1.2 *U*_eq_(C, N). The methyl H atoms are disordered and were refined in two different orientations.

### Crystal data

**Table:**

C_7_H_8_N_2_O	*Z* = 2
*M**_r_* = 136.15	*F*(000) = 144
Triclinic, *P*1	*D*_x_ = 1.282 Mg m^−^^3^
Hall symbol: -P 1	Mo *K*α radiation, λ = 0.71073 Å
*a* = 4.0611 (6) Å	Cell parameters from 23 reflections
*b* = 8.6232 (12) Å	θ = 8.8–16.8°
*c* = 10.3231 (12) Å	µ = 0.09 mm^−^^1^
α = 87.421 (12)°	*T* = 295 K
β = 79.344 (14)°	Plate, colorless
γ = 83.103 (15)°	0.5 × 0.2 × 0.1 mm
*V* = 352.61 (8) Å^3^	

### Data collection

**Table:**

Bruker P4 diffractometer	993 reflections with *I* > 2σ(*I*)
Radiation source: fine-focus sealed tube	*R*_int_ = 0.031
graphite	θ_max_ = 25.0°, θ_min_ = 4.6°
ω scans	*h* = −4→1
Absorption correction: ψ scan (*XSCANS*; Siemens, 1995)	*k* = −10→10
*T*_min_ = 0.713, *T*_max_ = 0.940	*l* = −12→12
1757 measured reflections	3 standard reflections every 97 reflections
1222 independent reflections	intensity decay: none

### Refinement

**Table:**

Refinement on *F*^2^	Primary atom site location: structure-invariant direct methods
Least-squares matrix: full	Secondary atom site location: difference Fourier map
*R*[*F*^2^ > 2σ(*F*^2^)] = 0.050	Hydrogen site location: inferred from neighbouring sites
*wR*(*F*^2^) = 0.148	H-atom parameters constrained
*S* = 1.05	*w* = 1/[σ^2^(*F*_o_^2^) + (0.0874*P*)^2^ + 0.0372*P*] where *P* = (*F*_o_^2^ + 2*F*_c_^2^)/3
1222 reflections	(Δ/σ)_max_ < 0.001
92 parameters	Δρ_max_ = 0.15 e Å^−^^3^
0 restraints	Δρ_min_ = −0.16 e Å^−^^3^

### Special details

**Table:**

Experimental. Refinement of *F*^2^ against ALL reflections. The weighted *R*-factor *wR* and goodness of fit *S* are based on *F*^2^, conventional *R*-factors *R* are based on *F*, with *F* set to zero for negative *F*^2^. The threshold expression of *F*^2^ > σ(*F*^2^) is used only for calculating *R*-factors(gt) *etc*. and is not relevant to the choice of reflections for refinement. *R*-factors based on *F*^2^ are statistically about twice as large as those based on *F*, and *R*- factors based on ALL data will be even larger.
Geometry. All e.s.d.'s (except the e.s.d. in the dihedral angle between two l.s. planes) are estimated using the full covariance matrix. The cell e.s.d.'s are taken into account individually in the estimation of e.s.d.'s in distances, angles and torsion angles; correlations between e.s.d.'s in cell parameters are only used when they are defined by crystal symmetry. An approximate (isotropic) treatment of cell e.s.d.'s is used for estimating e.s.d.'s involving l.s. planes.
Refinement. Refinement of *F*^2^ against ALL reflections. The weighted *R*-factor *wR* and goodness of fit *S* are based on *F*^2^, conventional *R*-factors *R* are based on *F*, with *F* set to zero for negative *F*^2^. The threshold expression of *F*^2^ > σ(*F*^2^) is used only for calculating *R*-factors(gt) *etc*. and is not relevant to the choice of reflections for refinement. *R*-factors based on *F*^2^ are statistically about twice as large as those based on *F*, and *R*- factors based on ALL data will be even larger.

### Fractional atomic coordinates and isotropic or equivalent isotropic displacement parameters (Å^2^)

**Table:**

	*x*	*y*	*z*	*U*_iso_*/*U*_eq_	Occ. (<1)
O	1.4562 (3)	0.34810 (14)	0.62540 (13)	0.0766 (5)	
N1	1.1428 (3)	0.58235 (15)	0.62843 (12)	0.0528 (4)	
H1A	1.2438	0.6042	0.5501	0.063*	
N2	0.7461 (3)	0.66352 (15)	0.81236 (13)	0.0509 (4)	
C1	0.3445 (5)	0.7290 (3)	1.01096 (18)	0.0728 (6)	
H1B	0.3774	0.6176	1.0235	0.109*	0.50
H1C	0.1076	0.7634	1.0212	0.109*	0.50
H1D	0.4388	0.7780	1.0751	0.109*	0.50
H1E	0.2384	0.8217	1.0564	0.109*	0.50
H1F	0.5083	0.6759	1.0587	0.109*	0.50
H1G	0.1771	0.6613	1.0048	0.109*	0.50
C2	0.5164 (4)	0.77275 (19)	0.87483 (16)	0.0553 (5)	
C3	0.4394 (5)	0.9158 (2)	0.8175 (2)	0.0685 (5)	
H3A	0.2829	0.9906	0.8637	0.082*	
C4	0.5969 (5)	0.9474 (2)	0.6904 (2)	0.0717 (6)	
H4A	0.5462	1.0435	0.6498	0.086*	
C5	0.8283 (4)	0.8360 (2)	0.62478 (18)	0.0609 (5)	
H5A	0.9351	0.8536	0.5385	0.073*	
C6	0.8977 (4)	0.69680 (18)	0.69102 (15)	0.0480 (4)	
C7	1.2323 (4)	0.4432 (2)	0.67961 (16)	0.0621 (5)	
H7A	1.1165	0.4164	0.7624	0.075*	

### Atomic displacement parameters (Å^2^)

**Table:**

	*U*^11^	*U*^22^	*U*^33^	*U*^12^	*U*^13^	*U*^23^
O	0.0884 (9)	0.0587 (8)	0.0646 (8)	0.0094 (7)	0.0201 (7)	0.0038 (6)
N1	0.0586 (8)	0.0521 (8)	0.0424 (7)	−0.0075 (6)	0.0051 (6)	0.0000 (6)
N2	0.0505 (8)	0.0535 (8)	0.0467 (7)	−0.0083 (6)	−0.0011 (6)	−0.0051 (6)
C1	0.0666 (11)	0.0840 (13)	0.0597 (11)	−0.0036 (9)	0.0098 (9)	−0.0148 (9)
C2	0.0468 (9)	0.0588 (9)	0.0589 (10)	−0.0068 (7)	−0.0031 (7)	−0.0124 (8)
C3	0.0574 (10)	0.0579 (10)	0.0857 (13)	−0.0004 (8)	−0.0027 (9)	−0.0137 (9)
C4	0.0680 (11)	0.0529 (10)	0.0918 (14)	−0.0039 (8)	−0.0123 (10)	0.0086 (9)
C5	0.0616 (10)	0.0566 (10)	0.0630 (10)	−0.0121 (8)	−0.0061 (8)	0.0095 (8)
C6	0.0463 (8)	0.0500 (9)	0.0479 (8)	−0.0112 (7)	−0.0046 (6)	−0.0036 (7)
C7	0.0702 (11)	0.0563 (10)	0.0493 (9)	−0.0014 (8)	0.0121 (8)	0.0035 (7)

### Geometric parameters (Å, °)

**Table:**

O—C7	1.2192 (19)	C1—H1F	0.9600
N1—C7	1.327 (2)	C1—H1G	0.9600
N1—C6	1.402 (2)	C2—C3	1.371 (3)
N1—H1A	0.8600	C3—C4	1.380 (3)
N2—C6	1.325 (2)	C3—H3A	0.9300
N2—C2	1.339 (2)	C4—C5	1.368 (3)
C1—C2	1.502 (2)	C4—H4A	0.9300
C1—H1B	0.9600	C5—C6	1.380 (2)
C1—H1C	0.9600	C5—H5A	0.9300
C1—H1D	0.9600	C7—H7A	0.9300
C1—H1E	0.9600		
			
C7—N1—C6	125.62 (13)	H1D—C1—H1G	141.1
C7—N1—H1A	117.2	H1E—C1—H1G	109.5
C6—N1—H1A	117.2	H1F—C1—H1G	109.5
C6—N2—C2	117.87 (15)	N2—C2—C3	122.02 (16)
C2—C1—H1B	109.5	N2—C2—C1	116.18 (15)
C2—C1—H1C	109.5	C3—C2—C1	121.80 (16)
H1B—C1—H1C	109.5	C2—C3—C4	119.19 (17)
C2—C1—H1D	109.5	C2—C3—H3A	120.4
H1B—C1—H1D	109.5	C4—C3—H3A	120.4
H1C—C1—H1D	109.5	C5—C4—C3	119.38 (17)
C2—C1—H1E	109.5	C5—C4—H4A	120.3
H1B—C1—H1E	141.1	C3—C4—H4A	120.3
H1C—C1—H1E	56.3	C4—C5—C6	117.69 (17)
H1D—C1—H1E	56.3	C4—C5—H5A	121.2
C2—C1—H1F	109.5	C6—C5—H5A	121.2
H1B—C1—H1F	56.3	N2—C6—C5	123.81 (16)
H1C—C1—H1F	141.1	N2—C6—N1	117.00 (14)
H1D—C1—H1F	56.3	C5—C6—N1	119.19 (14)
H1E—C1—H1F	109.5	O—C7—N1	124.40 (15)
C2—C1—H1G	109.5	O—C7—H7A	117.8
H1B—C1—H1G	56.3	N1—C7—H7A	117.8
H1C—C1—H1G	56.3		

### Hydrogen-bond geometry (Å, °)

**Table:**

*D*—H···*A*	*D*—H	H···*A*	*D*···*A*	*D*—H···*A*
N1—H1A···O^i^	0.86	2.04	2.8971 (19)	172

Symmetry codes: (i) −*x*+3, −*y*+1, −*z*+1.
